# Restrained Th17 response and myeloid cell infiltration into the central nervous system by human decidua-derived mesenchymal stem cells during experimental autoimmune encephalomyelitis

**DOI:** 10.1186/s13287-016-0304-5

**Published:** 2016-03-17

**Authors:** Beatriz Bravo, Marta I. Gallego, Ana I. Flores, Rafael Bornstein, Alba Puente-Bedia, Javier Hernández, Paz de la Torre, Elena García-Zaragoza, Raquel Perez-Tavarez, Jesús Grande, Alicia Ballester, Sara Ballester

**Affiliations:** Instituto de Salud Carlos III, Unidad Funcional de Investigación en Enfermedades Crónicas, Laboratory of Gene Regulation, Carretera de Majadahonda-Pozuelo Km 2, 28220 Madrid, Spain; Instituto de Salud Carlos III, Unidad Funcional de Investigación en Enfermedades Crónicas, Laboratory of Mammary Gland Pathology, Carretera de Majadahonda-Pozuelo Km 2, 28220 Madrid, Spain; Grupo de Medicina Regenerativa, Instituto de Investigación Hospital 12 de Octubre, Avda. Córdoba s/n, 28041 Madrid, Spain; Hospital Central de Cruz Roja, Servicio de Hematología y Hemoterapia, Avenida de Reina Victoria 24, 28003 Madrid, Spain; Instituto de Salud Carlos III, Unidad Funcional de Investigación en Enfermedades Crónicas, Histology Core Unit, Carretera de Majadahonda-Pozuelo Km 2, 28220 Madrid, Spain

**Keywords:** Placental mesenchymal stem cells, Th17, EAE, Immunomodulation

## Abstract

**Background:**

Multiple sclerosis is a widespread inflammatory demyelinating disease. Several immunomodulatory therapies are available, including interferon-β, glatiramer acetate, natalizumab, fingolimod, and mitoxantrone. Although useful to delay disease progression, they do not provide a definitive cure and are associated with some undesirable side-effects. Accordingly, the search for new therapeutic methods constitutes an active investigation field. The use of mesenchymal stem cells (MSCs) to modify the disease course is currently the subject of intense interest. Decidua-derived MSCs (DMSCs) are a cell population obtained from human placental extraembryonic membranes able to differentiate into the three germ layers. This study explores the therapeutic potential of DMSCs.

**Methods:**

We used the experimental autoimmune encephalomyelitis (EAE) animal model to evaluate the effect of DMSCs on clinical signs of the disease and on the presence of inflammatory infiltrates in the central nervous system. We also compared the inflammatory profile of spleen T cells from DMSC-treated mice with that of EAE control animals, and the influence of DMSCs on the in vitro definition of the Th17 phenotype. Furthermore, we analyzed the effects on the presence of some critical cell types in central nervous system infiltrates.

**Results:**

Preventive intraperitoneal injection of DMSCs resulted in a significant delay of external signs of EAE. In addition, treatment of animals already presenting with moderate symptoms resulted in mild EAE with reduced disease scores. Besides decreased inflammatory infiltration, diminished percentages of CD4^+^IL17^+^, CD11b^+^Ly6G^+^ and CD11b^+^Ly6C^+^ cells were found in infiltrates of treated animals. Early immune response was mitigated, with spleen cells of DMSC-treated mice displaying low proliferative response to antigen, decreased production of interleukin (IL)-17, and increased production of the anti-inflammatory cytokines IL-4 and IL-10. Moreover, lower RORγT and higher GATA-3 expression levels were detected in DMSC-treated mice. DMSCs also showed a detrimental influence on the in vitro definition of the Th17 phenotype.

**Conclusions:**

DMSCs modulated the clinical course of EAE, modified the frequency and cell composition of the central nervous system infiltrates during the disease, and mediated an impairment of Th17 phenotype establishment in favor of the Th2 subtype. These results suggest that DMSCs might provide a new cell-based therapy for the control of multiple sclerosis.

**Electronic supplementary material:**

The online version of this article (doi:10.1186/s13287-016-0304-5) contains supplementary material, which is available to authorized users.

## Background

Multiple sclerosis (MS) is a progressive inflammatory disorder of the central nervous system (CNS) elicited by an immune reaction against self-neuroantigens. Compelling support for the autoimmune etiology of MS is provided by the experimental autoimmune encephalomyelitis (EAE) animal model of CNS inflammation. In this model, illness is triggered by the immune response to experimentally supplied auto-antigens [1]. This reaction is driven by auto-responsive T cells in lymph nodes and spleen able to migrate to the CNS and cross the blood–brain barrier, where they find their cognate antigen in the context of resident antigen-presenting cells, such as microglia or astrocytes, or of immigrant macrophages or dendritic cells. These events favor an inflammatory environment with CNS injury, characterized by loss of the insulating myelin sheath of neuronal axons resulting in motor disability. EAE can also be induced by transplanting T helper (Th) cells delivering interleukin (IL)-17 (Th17) or interferon (IFN)-γ (Th1) [2]. Since IFN-γ has, however, demonstrated a dual role in disease pathogenesis, the IL-17 pathway is considered a more appropriate therapeutic target [3, 4]. Two other subsets of CD4^+^ cells, namely Th2 and T regulatory (Treg) cells, are able to control or ameliorate EAE disease evolution [5–7] by secreting cytokines such as IL-4 [8], IL-10 [9], and transforming growth factor (TGF)-β [10]. Several other cytokines are able to modify EAE course, such as IL-27—a main negative regulator of Th17 development [11–14].

Although significant progress has been made in MS therapy, none of the available treatments achieves a halt or reversion of disability progression. Hence, development of new therapeutic strategies is a crucial challenge. To this end, stem cells have been introduced into the MS scenario in recent years. While some reports support possible advantages of embryonic over adult stem cells [15], ethical concerns about the use of the former promote the study of adult stem cells [16, 17]. Several phase I/II clinical trials underway in MS patients are evaluating the therapeutic potential of mesenchymal stem cells (MSCs) derived from different tissues, such as bone marrow (BM), adipose tissue, placental or umbilical cord blood [16–23]. Preliminary results indicate that administration of MSCs to patients with MS is feasible and safe. In addition, some studies have reported a degree of structural, functional, and physiological improvement after treatment, consistent with the immunomodulatory and neuroprotective effects of MSCs. Despite early clinical stabilization or improvement in some of these patients, further controlled trials are warranted to evaluate alternative cell sources and administration schedules which might affect MS disease course more consistently. Some preclinical data from experimental models would appear to supply grounds for postulating neuroprotective and immunomodulatory properties for MSCs. Neuroprotection by MSCs is suggested by reports showing stimulation of oligodendrogenesis, oligodendrocyte progenitor migration, remyelination, and reduction of axonal loss [24–27]. Some MSC subpopulations deliver active neurotrophins [28], and human neural progenitors obtained in vitro from MSCs improved neurological function in EAE [29]. Immunomodulatory effects ascribed to MSCs throughout EAE treatment include hepatocyte growth factor production [30], prostaglandin E2 secretion [31], promotion of IL-27 [14], inhibition of IL-17 and tumor necrosis factor-alpha (TNFα) production [24, 32], downregulation of IFNγ T-cell expression [33] and T-cell anergy [34].

Most of the studies focused on adult MSC therapy for EAE have used BM as a cell source. However, MSCs derived from other tissues, such as adipose [35, 36], endometrial [37], umbilical cord [38, 39], or placenta [40, 41], have also been shown to influence EAE development. MSC content in adult BM is limited, invasive procedures are required for MSC procurement, and the number and differentiation capacity of such cells decrease with the age of the donor. Placenta-derived MSCs present several advantages over other sources, since the cells can be isolated without any donor injury and a large amount of cells with high differentiation ability can be obtained [42]. We previously characterized a subset of human decidua-derived MSCs (DMSCs) with capacity to differentiate at the clonal level into the three embryonic germ layers [42–45]. DMSCs display some properties of embryonic cells and others of adult stem cells, as they express transcription factors involved in pluripotency (Oct-4 and Rex-1) and organogenesis (GATA-4), though not embryonic markers (SSEA-1, -3, -4 and TRA-1-81) [42] expressed by other placental-derived MSCs [46]. DMSCs are of maternal origin and show higher proliferation rates and differentiation capacity than do BM-derived MSCs [47, 48], thereby making them biologically different to MSCs derived from other adult sources.

In the present study we have evaluated the therapeutic potential of DMSCs on EAE. Results showed that a prophylactic treatment with DMSCs was able to delay the onset and reduce the severity of the disease substantially for as long as treatment was maintained. Furthermore, the therapeutic utility of DMSCs was also demonstrated in animals which were initially treated when they presented with moderate symptoms, with this resulting in a mild course of EAE. DMSC treatment reduced CNS injury areas and modulated the peripheral immune response, leading to an anti-inflammatory profile of spleen T cells. The frequency and cell composition of CNS infiltrates were also modified, with the percentages of CD4^+^IL-17^+^, CD11b^+^Ly6G^+^, and CD11b^+^Ly6C^+^ cells being reduced by DMSC treatment.

## Methods

### Animals, EAE induction, clinical evaluation and treatments

All experiments were conducted under institutional ethical and safety guidelines, with approval number 017/15 of the Madrid Regional Authority’s Ethics Committee, in accordance with European Union legislation. C57BL/6 mice were bred and maintained at the Institution’s animal facility. Mice were housed in groups of 4–5. To induce EAE, 10- to 14-week-old female mice were anesthetized by intraperitoneal administration of ketamine and xylazine and immunized by subcutaneous injection in flanks with 200 μg myelin oligodendrocyte glycoprotein (MOG)_35–55_ peptide (Peptide 2.0, Chantilly, VA, USA) in complete Freund adjuvant containing 2.5 mg/ml *Mycobacterium tuberculosis* H37RA (Difco) in a total volume of 100 μl. *Bordetella pertussis* toxin (300 ng in 100 μl) was administered intraperitoneally on the day of antigen inoculation and 48 hours later (D0 and D2 post-immunization (p.i.), respectively). Groups of 7–10 animals were used for each experiment. Clinical signs were scored on a 0–5 scale as follows: no clinical signs, 0; loss of tail tonicity, 1; rear limb weakness, 2; paralysis of one rear limb, 3; paralysis of two rear limbs, 4; full paralysis of four limbs, 5. At value 4, animals were sacrificed to avoid further progress of the disease. Score values were calculated as the average of the evaluations assigned to each mouse by three independent observers in blind inspection. For DMSC treatments, cells at passage 6–8 with 95–98 % viability were used. At this passage number, the cells still preserve a high proliferation and multilineage differentiation capacity [42]. One million cells were administered in 100 μl phosphate-buffered saline (PBS) by intraperitoneal injection to every treated animal on the days indicated for each experiment.

### Isolation of human DMSCs and culture

Human placentas from healthy mothers were supplied by the Department of Obstetrics and Gynecology under written consent previously approved by the Ethics Committee at the Hospital Universitario 12 de Octubre. DMSC isolation and culture was performed as previously described [42]. Briefly, placental membranes were digested with trypsin-versene (Lonza, Spain), and the cells were seeded at 1.2 × 10^5^ cells/cm^2^ and cultured at 37 °C, 5 % CO_2_ and 95 % humidity in Dulbecco’s modified Eagle medium (DMEM; Lonza) supplemented with 2 mM L-glutamine, 0.1 mM sodium pyruvate, 55 μM B-mercaptoethanol, 1 % nonessential amino acids, 1 % penicillin/streptomycin, 10 % fetal bovine serum and 10 ng/ml epidermal growth factor 1 (EGF-1; Sigma-Aldrich Química, Spain). The morphology, phenotype and MSC characteristics of DMSCs have been previously reported [42]. Cells were cryopreserved and, before use, were thawed and passaged at a density of around 5 × 10^4^ cells/cm^2^ until passage 6–8.

### Mouse cell isolation and culture

Mouse spleen cells were obtained as previously described [49]. CD4^+^ cells were magnetically sorted (Miltenyi Biotech) to 90–95 % purity, and tested by flow cytometry with anti-CD4 antibody (L3T4; Miltenyi Biotech). Total spleen population or purified CD4^+^ cells from each group of animals were pooled, washed and suspended in Click’s medium [50] before in vitro culture. For anti-CD3/anti-CD28 stimulation, cells were cultured in microwell plates coated with anti-CD3 (Y-CD3-1, 10 μg/ml) [51] and soluble anti-CD28 (clone 37.51, 1 ng/ml; eBioscience, Hatfield, UK). For antigenic stimulation, 25 μM MOG_35–55_ was used in cell cultures. Th17 phenotype skewing conditions were achieved by IL-6 and TGFβ treatment as previously described [52]. Briefly, anti-CD3/anti-CD28 stimulation was supplemented with 20 ng/ml IL-6 (eBioscience), 5 ng/ml TGFβ (eBioscience), 25 μg/ml anti-IL-4 (11B11; ATCC HB188) and 25 μg/ml anti-IFN-γ (R46A2; ATCC HB170). Cocultures of DMSC-murine spleen cells were performed at a ratio of 1:7. First, plates were seeded with DMSCs in DMEM supplemented with EGF-1 (10 ng/ml; Sigma-Aldrich Química). After 12 hours this medium was removed and spleen cells were added in Click’s medium with soluble anti-CD3 (25 μg/ml) and anti-CD28 (1 μg/ml). For isolation of CNS inflammatory infiltrates, animals were sacrificed and perfused through the left ventricle with 200 ml PBS to wash out leukocytes present within the blood vessels. Spinal cords and brains were removed, and tissue from each mouse was homogenized through a 100-μm pore strainer. After centrifugation, the pellet was dissolved in 30 % Percoll (Amersham) and the homogenate mix was layered over 80 % Percoll. Infiltrating cells were collected from the 30–80 % interface, after centrifugation at 3,000 rpm for 30 minutes at room temperature without brake. Spleen cells for early immune response analysis were obtained from animals at day 7–10 p.i., while CNS studies were performed on mice at day 20 p.i.

### T-cell proliferation and cytokine expression measurements

Total spleen cells or purified CD4^+^ cells (2 × 10^5^) were split into p-96 well microtiter and subjected to T lymphocyte-specific stimuli (anti-CD3/anti-CD28 antibodies or MOG_35–55_, as specified above) through 72 hours. Colorimetric assay based on MTT was used as a T-cell proliferation measurement according to that described in [53]. Cytokines released to the medium were quantified by enzyme-linked immunosorbent assay (ELISA), with each sample assayed in quintuplicate. Capture and biotin-conjugated antibodies were, respectively: eBio17CK15A5 and eBio17B7 (e-Bioscience) for IL-17A; Jes5-2A5 and Jes5-16E3 (BD-Bioscience) for IL-10; and 11B11 and BVD6-24G2 (Becton-Dickinson) for IL-4. Obtention of total RNA for retrotranscription and quantitative real-time polymerase chain reaction (RT-qPCR) were performed as previously reported [54]. The primer pairs used for each gene were as follows:

- IL-17 forward: 5′-GAAGCTCAGTGCCGCCA-3′;
- IL-17 reverse: 5′-TTCATGTGGTGGTCCAGCTTT-3′;
- IL-4 forward: 5′-ATCCTGCTCTTCTTTCTCG-3′;
- IL-4 reverse: 5′-GATGCTCTTTAGGCTTTCC-3′;
- IL-10 forward: 5′-TGCTATGCTGCCTGCTCTTA-3′;
- IL-10 reverse: 5′-GCTCCACTGCCTTGCTCTTA-3′;
- RORγT forward: 5′-CCGCTGAGAGGGCTTCAC-3′;
- RORγT reverse: 5′-TGCAGGAGTAGGCCACATTACA-3′;
- GATA-3 forward: 5′-AGAACCGGCCCCTTATCAA-3′;
- GATA-3 reverse: 5′-AGTTCGCGCAGGATGTCC-3′;
- Foxp3 forward: 5′-ACCACCTTCTGCTGCCACTG-3′;
- Foxp3 reverse: 5′-TGCTGTCTTTCCTGGGTGTACC-3′;
- β-actin forward: 5′-TGTTACCAACTGGGACGACA-3′; and,
- β-actin reverse: 5′-GGGGTGTTGAAGGTCTCAAA-3′.

PCR product quality was checked by a melting curve analysis for each sample and the reaction efficiencies were checked to be near 2. Each result was normalized by the housekeeping β-actin gene expression. Relative quantification of gene expression analysis was performed using the Pfaffl method [55].

### Flow cytometry cell staining

For all experiments, cells were incubated in 0.5 μg FcBlock (BD Bioscience) for 10 minutes at room temperature. Surface molecule staining was performed in the dark for 30 minutes at 4 °C. Cells were then washed twice with staining buffer followed by fixation in 1 % paraformaldehyde. Antibodies for surface markers were: anti-CD4 clone RM4-5 biotin (eBioscience); anti-CD8α clone 53–6.7 biotin (eBioscience); anti-CD19 clone 1D3 biotin (eBioscience); anti-NK1.1 clone PK136 biotin (eBioscience); anti-CD11c clone HL3 biotin (BD-Pharmingen); anti-CD11b clone M1/70 APC (eBioscience); anti-Ly6G clone AL-21 APC-Cy7 (BD-Pharmingen); and anti-Ly6C clone 1A8 PE (BD-Pharmingen). For biotin antibodies, streptavidin PE-Cy7 (eBioscience) was used to detect positive cells. For intracellular staining, anti-IL17 (clone TC11-18H10-PE; BD-Pharmingen), anti-RORγT (clone AFKJS-9-APC; eBioscience) and anti-Foxp3 (clone FJK-16S; eBioscience) were used on cells previously permeabilized and fixed by Cytofix/CytopermTM (Becton Dickinson) and Staining Set Kit (eBioscience), respectively, for cytoplasmic IL-17 and nuclear RORγT detection. Cells were acquired on a BD FACSCantoTM II. Data were collected by BD FACS Diva software and analyzed by FlowJo software (Tree Star Inc.). Fluorescence minus one (FMO) controls were used for gating analysis to distinguish positive and negative cell populations. Propidium iodide staining was used for live/dead discrimination. Compensation was carried out using single color controls, and compensation matrices were calculated and applied by FlowJo software.

### Histopathology

Mice were anesthetized by intraperitoneal administration of ketamine-xylazine and transcardially perfused with 4 % paraformaldehyde. Spinal cords and brains were fixed in 4 % paraformaldehyde. Vibratome free-floating slices (15–30 μm) were preserved in 0.1 M phosphate buffer. For detection of demyelinating and inflammatory lesions, slices were subjected to luxol fast blue (LFB)–periodic acid-Schiff (PAS)–hematoxylin triple staining according to Goto [56] and hematoxylin–eosin staining, performed as previously described [52]. Perivascular infiltrates were quantified by examining hematoxylin–eosin serial sections along the brain and spinal cord of each animal. Slices were classified as positive or negative for infiltrate quantification. For immunodetection of CD4^+^ and GFAP^+^ cells, free-floating spinal cord sections were boiled in a microwave oven in 10 mM sodium citrate buffer for antigen retrieval. Prior to incubation with antibodies, endogenous peroxidase activity was inhibited with 2 % hydrogen peroxide in CD4 immunohistochemistry samples, and tissue autofluorescence was minimized by 2 % sodium borohydride treatment of the immunofluorescence samples. As primary antibodies, L3T4 (Sino Biological Inc), EPR1034Y (Millipore) and PC10 (Abcam) were, respectively, used for detection of CD4, GFAP and PCNA (as a marker of cell proliferation). Overnight incubation with primary antibody was followed by 1-hour incubation with Biotin-conjugated goat anti-rabbit (Jackson Inmunoresearch Lab Inc.) for CD4 detection, or Alexa Fluor 594 Donkey anti-rabbit IgG (Invitrogen) and Alexa Fluor 488 Donkey anti-mouse IgG (Invitrogen) for immunofluorescence of GFAP and PCNA, respectively. Thereafter, samples for CD4 immunohistochemistry were exposed for 30 minutes to Vectastain ABC reagent (Vector Laboratories) and to DAB developing solution (Vector Laboratories), and counterstained with hematoxylin for visualization using a Leica DM2000 microscope. Fluorescent images were captured by confocal microscopy using a Leica TCS SP5 AOBS Confocal Microscope (Leica Microsystems GmbH, Wetzlar, Germany), and analysis was performed with Image J software designed by the NIH (MD, USA). In all cases, specificity of staining was confirmed by controls omitting the primary antibody.

### Statistics

Statistical analyses were performed with Graph Pad Prism version 5.02 (Graph Pad software, Inc). The *t*-test was used for unpaired data; in cases where n ≤ 10 (with a minimum of 5), Welch’s correction was introduced in order not to assume equal variances. Contingency table analysis for comparison of perivascular infiltrate quantification and disease incidence was performed using Chi-square test (n > 30) or Fisher’s test (n < 30, with a minimum of 14). The area under the curve (AUC) was calculated from EAE clinical course for each mouse, and differences between groups were analyzed by the Mann–Whitney test. Statistical significance is indicated as *p < 0.05, **p < 0.01 or ***p < 0.001.

## Results

### DMSC treatment delays the development of EAE and restrains early Th17 response

**Fig. 1:**
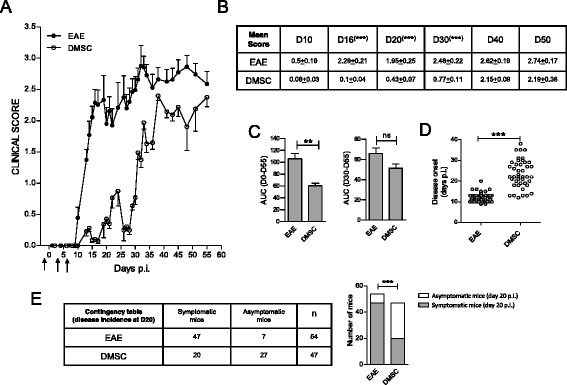
Decidua-derived mesenchymal stem cell (*DMSC*) treatment delays onset of MOG-experimental autoimmune encephalomyelitis (*EAE*). Groups of 7–10 C57BL/6 mice were established by MOG_35–55_ peptide inoculation. **a** DMSC group animals received 1 × 10^6^ DMSCs at days −1, 3 and 6 post-immunization (*p.i.*) (*arrows*). Daily mean clinical scores along EAE course are shown from one representative (n = 10 mice/group) out of five independent experiments. **b** Statistical significance by unpaired *t*-test for mean scores at different days after immunization. **c** Area under the curve (*AUC*) was calculated from EAE clinical course for each mouse between days 0 and 55 (D0–55) or between days 30 and 55 p.i. (D30–D50), and differences between groups were analyzed using the Mann–Whitney test. Standard error of the means are shown. Data from individual mice included in five independent experiments were used to examine **d** individual disease onset and **e** disease incidence at day 20 p.i.; the differences between groups were analyzed by *t*-test and by Chi-square test, respectively (n for each group is indicated). The bar graphs for representation of the disease incidence contingency table show the numbers of symptomatic and asymptomatic mice at day 20 p.i. for each group. **p < 0.01, ***p < 0.001. *ns* Not significant

To determine whether DMSC administration had protective effects on EAE, we designed a preventive approach comprising three intraperitoneal injections of cells at days −1, 3 and 6, with the day of MOG inoculation being established as day 0. Daily monitoring of score values showed that DMSC treatment resulted in a significant delay in the onset of EAE symptoms. The first clinical signs were apparent in the EAE control group at day 10–13 p.i., whereas most of the DMSC-treated mice did not show symptoms of established disease (score values higher than 1) before day 25–30 p.i. (Fig. 1a). Although DMSC-treated animals ultimately attained score values near those of the EAE control group, significant differences between both groups of mice remained until at least day 30 p.i. (Fig. 1b). We also evaluated the AUC as a measure of disease severity. When such analysis was restricted to the late phase of the clinical course (from day 30 to day 55 p.i.), no difference was found between groups. However, overall clinical course examination showed significantly lower values for the DMSC group, in line with the beneficial effect of DMSCs until at least day 30 p.i. (Fig. 1c). Furthermore, data from individual mice demonstrated notably delayed disease onset (Fig. 1d) and decreased disease incidence evaluated at day 20 p.i. (Fig. 1e) for DMSC-treated animals with respect to the EAE group.

**Fig. 2:**
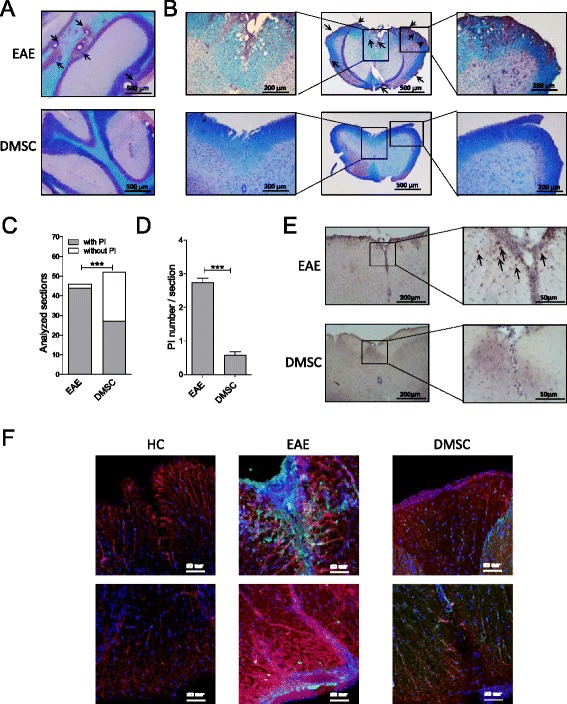
Decidua-derived mesenchymal stem cell (*DMSC*) treatment of experimental autoimmune encephalomyelitis (*EAE*) decreases inflammation in the CNS. **a** Cerebellum and **b** spinal cord sections from EAE control and DMSC-treated animals were LFB–PAS–hematoxylin stained; *arrows* show perivascular infiltrates (*PI*). Hematoxylin–eosin stained slices were classified as positive (with PI) or negative (without PI) for presence of perivascular infiltrates; the difference between the EAE group (n = 47) and the DMSC group (n = 52) was analyzed by Chi-square test and shown as the histogram representation of the contingency table (**c**). **d** Each section was also classified according to the number of PI that they contained and the averages of PI/section for each group were compared by *t*-test; standard error of the means are shown. Immunohistochemistry with **e** anti-CD4 antibody (*arrows* show CD4^+^ cells) and **f** immunofluorescence for astrocytes with anti-GFAP (*red*), anti-PCNA (*green*) and DAPI (*blue*) are illustrative. Scale bars for magnifications are indicated. ***p < 0.001. *HC* Healthy control, *EAE* Untreated EAE group, *DMSC* DMSC-treated group

For a more in-depth examination of differences between DMSC-treated and untreated animals, we chose the disease phase with the most striking divergences in clinical signs (days 10–20 after MOG inoculation). Histopathological images of cerebellum and spinal cord sections from animals sacrificed at day 20 p.i. supported that DMSCs attenuated CNS pathology in EAE. LFB–PAS–hematoxylin staining showed important areas of myelin disruption in EAE mice whereas DMSC administration contributed to preservation of myelin integrity (Fig. 2a and b). In addition, DMSC-treated mice showed a smaller degree of infiltration, as both the number of analyzed hematoxylin–eosin stained sections showing perivascular infiltration (Fig. 2c) and the number of perivascular infiltrates per section (Fig. 2d) were strongly reduced in DMSC-treated mice. Moreover, CD4^+^ cells were frequent in the infiltration areas of EAE animals, while CD4 immunoreactivity was rather limited in DMSC-treated mice (Fig. 2e). Likewise, GFAP staining of spinal cord from EAE animals showed swollen astrocytic processes, indicative of astrocytic reactivity, with less severity in DMSC-treated mice (Fig. 2f). Furthermore, the use of anti-PCNA as a marker of cell proliferation revealed a lessening of astrocyte-division activity by DMSC treatment.

**Fig. 3:**
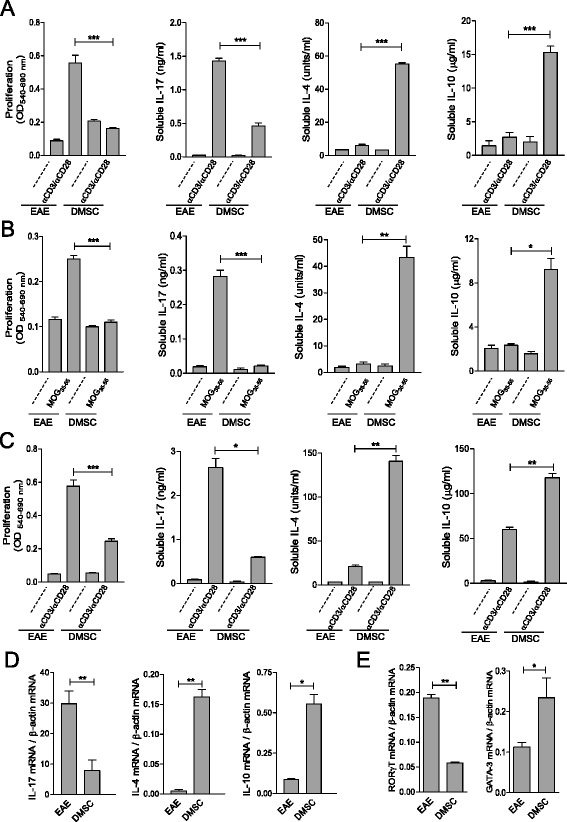
Decidua-derived mesenchymal stem cell (*DMSC*) treatment of experimental autoimmune encephalomyelitis (*EAE*) promotes anti-inflammatory T-cell profile. Total spleen cell population (**a, b**) or purified CD4^+^ cells (**c**) from EAE animals were obtained at day 10 p.i. Cells from each group were pooled and stimulated in vitro by anti-CD3 and anti-CD28 antibodies or by MOG_35–55_ peptide as indicated; *dashed lines* designate unstimulated cell cultures. Each sample was assayed in quintuplicate. Proliferation and soluble cytokines released to the medium were measured by the MTT colorimetric method and ELISA, respectively. RNA from anti-CD3/anti-CD28-stimulated CD4^+^ cells was used for RT-qPCR reactions to evaluate mRNA expression levels of cytokines (**d**) or RORγT and GATA-3 transcription factors (**e**). Results are shown from one representative out of five independent experiments. Significance was analyzed by *t*-test; standard error of the means are shown. *p < 0.05, **p < 0.01, ***p < 0.001. *IL* Interleukin, *MOG* Myelin oligodendrocyte glycoprotein, *OD* Optical density

We next studied the early immune response in both groups of animals by comparing T-cell reactivity in total spleen cell populations from DMSC-treated and untreated EAE mice shortly after MOG_35–55_ inoculation (day 7–10 p.i.). Cells were stimulated by anti-CD3 and anti-CD28 antibodies to induce broad T-lymphocyte activation, and by MOG_35–55_ to induce antigen-specific T-cell reactivity. In both cases, cells from DMSC-treated mice showed reduced proliferation and IL-17 release as compared to EAE control mice, while expression of the anti-inflammatory cytokines IL-4 and IL-10 were markedly increased in cultures of cells from DMSC-treated animals as against control (Fig. 3a and b). These results were also observed when purified CD4^+^ cell fractions were evaluated, suggesting a direct effect on this cell population without the need for other intermediate cell types (Fig. 3c). Moreover, analysis of IL-17, IL-4 and IL-10 mRNA levels from DMSC-treated versus untreated animals showed marked differences between the two groups which correlated with the results of soluble cytokine release (Fig. 3d). RORγT and GATA-3 transcription factors are known to be the master transcription factors that control the definition of Th17 and Th2 phenotypes, respectively [57]. We also analyzed their mRNA levels and found that, while RORγT expression was downregulated, GATA-3 mRNA levels were upregulated in CD4^+^ cells from DMSC-treated mice as compared to EAE untreated mice (Fig. 3e), which suggests that the Th17 phenotype is restrained while the Th2 subset is favored by DMSC treatment. Conversely, quantification of the mRNA levels of Foxp3, the transcription factor controlling Treg cell development, did not show differences between CD4^+^ cells from untreated and DMSC-treated EAE mice (Figure S1 in Additional file 1).

### Impaired establishment of Th17 phenotype in DMSC-treated animals is independent of experimentally induced inflammatory response

**Fig. 4:**
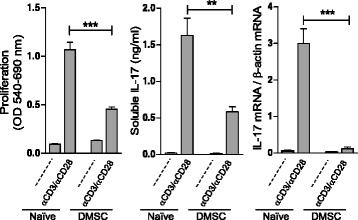
Decidua-derived mesenchymal stem cell (*DMSC*) administration to healthy mice reduces IL-17 production. Healthy C57BL/6 mice (5 mice/group) were exposed to three successive doses of DMSCs (*DMSC* group) or PBS (*Naïve* group) at 3-day intervals. Seven days after the first DMSC inoculation, total spleen cells from each group were pooled and stimulated in vitro by anti-CD3/anti-CD28. Each sample was assayed in quintuplicate. Results are shown from one representative out of three independent experiments. Proliferation, soluble IL-17 released to the medium, and IL-17 mRNA were measured by the MTT colorimetric method, ELISA and RT-qPCR, respectively. Significance was analyzed by *t*-test; standard error of the means are shown. **p < 0.01, ***p < 0.001. *IL* Interleukin, *OD* Optical density

To ascertain whether the effects of DMSC treatment on T-cell activity were dependent on the inflammatory response triggered by MOG inoculation, healthy mice were subjected to three successive DMSC doses every 3 days to emulate the treatment procedure used for EAE animals. As previously observed for EAE mice, T-cell proliferation and IL-17 expression were lower in DMSC-primed mice than in naïve animals (Fig. 4), suggesting that DMSCs do not require an inflammatory environment to downregulate the inflammatory potential of T cells.

**Fig. 5:**
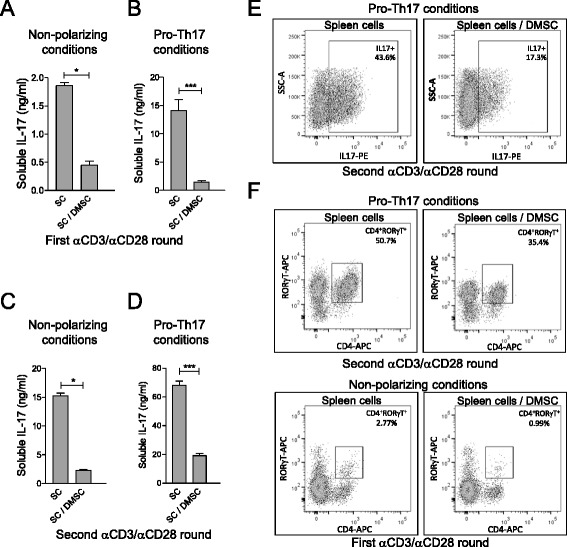
In vitro treatment of T cells with decidua-derived mesenchymal stem cells (*DMSC*) interferes with Th17 phenotype definition. Spleen cells (*SC*) from C57BL/6 mice were stimulated in vitro by anti-CD3/anti-CD28 antibodies under nonpolarizing condition (**a, c**) or under pro-Th17 pressure in the presence of IL-6 and TGFβ (**b, d, e**). Three-day cultures were analyzed for IL-17 expression (**a, b**) or used for a second round of anti-CD3/anti-CD28 stimulation after DMSC removal during 3 subsequent days before new analysis (**c, d, e**). Soluble IL-17 measurements were quantified by ELISA (**a–d**). Each sample was assayed in quintuplicate and significance was analyzed by *t*-test; standard error of the means are shown. Percentages of IL-17^+^ (**e**) or RORγT^+^ (**f**) cells were determined by FACS analysis of intracellular staining with anti-IL17 and anti-RORγT antibodies, respectively (**e**). Results are shown from one representative out of three independent experiments. *p < 0.05, ***p < 0.001. *IL* Interleukin, *Th* T helper

We next examined the influence of DMSCs on Th17 phenotype establishment in vitro. When spleen T cells and DMSCs were cocultured in vitro under nonpolarizing effector T cell condition (Fig. 5a) or under Th17-skewing condition in the presence of IL-6 and TGFβ (Fig. 5b), soluble IL-17 release was reduced as compared to cultures of exclusively spleen cells. The same results were obtained when the CD4^+^ cell population was cocultured with DMSCs (Figure S2 in Additional file 2), implying that, as in the case of in vivo treatments, there is a direct effect of DMSCs on CD4^+^.

This deficiency in IL-17 production seems to be acquired in perpetuity, since removal of DMSCs from the culture before a second round of T-cell stimulation was unable to restore the levels of IL-17 released by the T cells that had been previously cocultured with DMSCs (Fig. 5c and d). Accordingly, flow cytometry analysis of IL-17^+^ and RORγT^+^ cells showed a decrease in both Th17 cell markers expressed by the T cells cocultured with DMSCs (Fig. 5e and f).

**Fig. 6:**
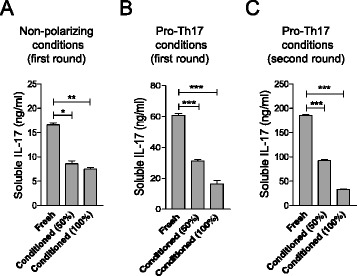
DMSC effect on Th17 phenotype definition is mediated by soluble factors. DMSC-culture supernatant from one EGF-1-free passage was used as conditioned medium for C57BL/6 spleen cells. Spleen cells were stimulated by anti-CD3 and anti-CD28 antibodies in fresh Click’s medium, in Click’s medium diluted one-half with conditioned medium (50 %), or in whole conditioned medium (100 %). Soluble IL-17 levels were evaluated by ELISA after 3 days under nonpolarizing condition (**a**), in the presence of IL-6 and TGF-β (**b**), or after a second round of anti-CD3/anti-CD28 stimulation in the absence of DMSC supernatant (**c**). Each sample was assayed in quintuplicate and significance was analyzed by *t*-test; standard error of the means are shown. Results are shown from one representative out of three independent experiments. Conditioned medium was supplemented with 10 % fresh fetal bovine serum before use. *p < 0.05, **p < 0.01, ***p < 0.001. *IL* Interleukin, *Th* T helper

To explore whether the effect of DMSCs on IL-17 production was dependent on a direct cell interaction between DMSCs and T cells, we used the supernatant of DMSC cultures as conditioned medium for anti-CD3/anti-CD28 T-cell stimulation. Inhibition of IL-17 secretion was consistently found for both nonpolarizing (Fig. 6a) and pro-Th17 (Fig. 6b) culture conditions, even after DMSC supernatant removal (Fig. 6c), indicating that cellular interaction is not required, but that one or more soluble factors produced by DMSCs are involved in IL-17 inhibition.

### Influence of DMSC treatment on the content of pro-inflammatory cell types in EAE CNS infiltrates

**Fig. 7:**
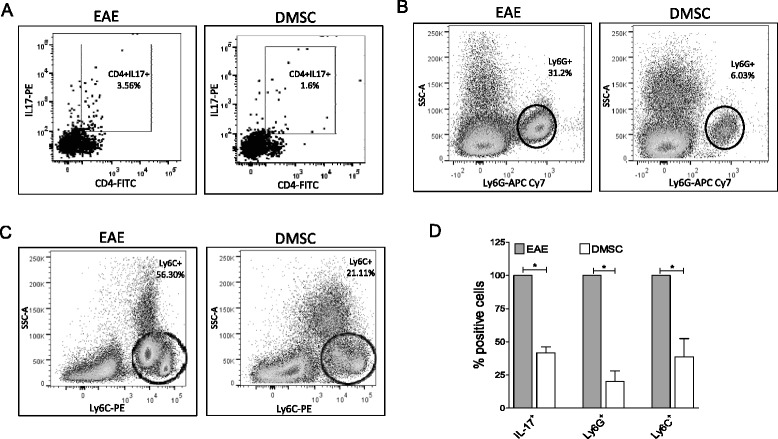
Decidua-derived mesenchymal stem cell (*DMSC*) treatment of experimental autoimmune encephalomyelitis (*EAE*) modifies the cell composition of inflammatory infiltrates in the CNS. CNS infiltrates from C57BL/6 EAE and DMSC-treated mice (n = 4/group) were obtained at day 20 p.i. and subjected to flow cytometry analysis. Debris and doublets were excluded and live/dead discrimination was determined using propidium iodide. Percentages of CD4^+^IL-17^+^ were obtained by analysis of surface and intracellular staining with anti-CD4 and anti-IL17, respectively (**a**). CD11b^+^ Lineage^-^ cells (gated out using CD4, CD8, CD19, CD11c, NK1.1 biotin-PE-Cy7 dump channel) were then subgated for identification of CD11b^+^Ly6G^+^ subpopulation (infiltrating neutrophils) (**b**) or CD11b^+^Ly6C^+^SSC^low^ subpopulation (infiltrating monocytes) (**c**) cells are shown by representative flow dot plots from two experiments. Data for quantification of infiltrating cell types in DMSC-treated mice are shown as percentages of each subset found in untreated mice (**d**). Significance was analyzed by *t*-test; standard error of the means are shown. *p < 0.05. *IL* Interleukin

The above data demonstrate that DMSCs can modulate both CD4^+^ T-cell activity and Th subset definition in lymphoid organs. To address whether such modulation correlates with the severity of inflammatory infiltration in the CNS, the content of different immune cell types with recognized inflammatory contribution was analyzed in CNS infiltrates. CD4^+^IL-17^+^ cell analysis showed that this cell type was present in a smaller percentage in DMSC-treated mice (Fig. 7a and d).

Two CD11b^+^ cell subtypes, Ly6G^+^ and Ly6C^+^SSC^low^, which identify neutrophils and inflammatory monocytes, respectively [58, 59], were also quantified. Treatment of EAE mice with DMSCs resulted in a reduction of infiltrating neutrophils (Fig. 7b and d), a significant component of immune infiltration in the CNS during EAE [60–62] whose recruitment is related to IL-17 activity [63]. In our experiments, CD11b^+^Ly6C^+^ inflammatory monocytes (other pathogenic players in inflammation [64–66]) could be divided into two subsets (Ly6C^int^ and Ly6C^high^), as described by Vainchtein et al. [67]. Both Ly6C^int^ and Ly6C^high^ cell percentages were lower in the infiltrates of the DMSC-treated group than in those of the control animals (Fig. 7c and d).

### Long-term DMSC treatment provides a more lasting therapeutic effect on EAE

**Fig. 8:**
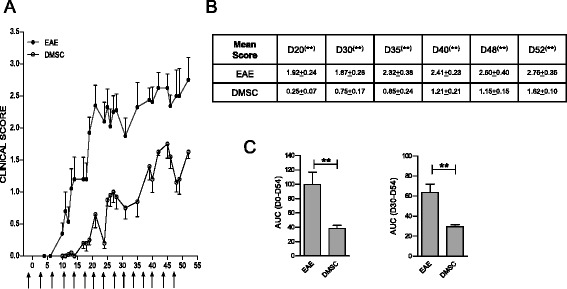
Decidua-derived mesenchymal stem cell (*DMSC*) continuous treatment sustains mild experimental autoimmune encephalomyelitis (*EAE*) course. **a** EAE mice were subjected to DMSC administration every 3–4 days, beginning the day before MOG_35–55_ injection (*arrows*). Daily mean clinical scores from one representative out of two independent experiments are shown. **b** Statistical significance by unpaired *t*-test for mean scores at different days after immunization. **c** Area under the curve (*AUC*) was calculated between days 0 and 54 (D0–54) or between days 30 and 54 p.i. (D30–D54), and differences between groups were analyzed using the Mann–Whitney test; standard error of the means are shown; n = 14 mice/group. **p < 0.01

All the above mentioned EAE experiments were performed following the stipulated preventive approach, based on three doses of DMSCs at days −1, 3 and 6 with respect to the day of MOG inoculation. We wished to investigate further the effects of DMSCs on EAE by continuous dosages of DMSCs over a longer period after MOG inoculation (Fig. 8a). In contrast to the result yielded by the brief treatment used beforehand, extended administration of DMSCs showed significant differences in clinical scores between untreated and DMSC-treated groups for at least 52 days after MOG_35–55_ inoculation (Fig. 8b). In addition, disease severity as measured by the AUC was significantly lower for the DMSC group, even at advanced phases of the clinical course of the disease (Fig. 8c).

**Fig. 9:**
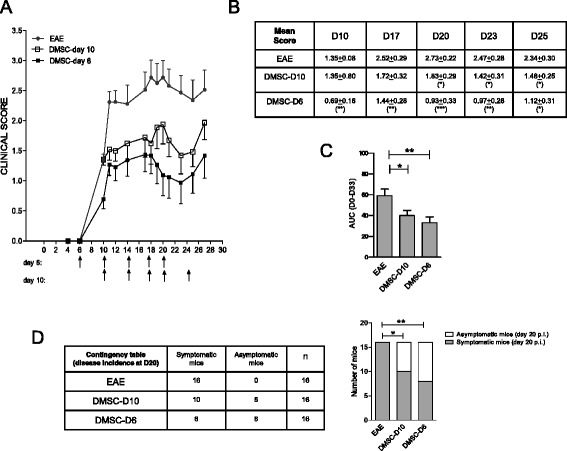
Decidua-derived mesenchymal stem cell (*DMSC*) treatment after experimental autoimmune encephalomyelitis (*EAE*) triggering. **a** EAE mice were subjected to DMSC administration every 3–4 days, beginning at day 6 (D6) or at day 10 (D10) after MOG_35–55_ injection (*arrows*). Daily mean clinical scores from one representative out of two independent experiments are shown. **b** Statistical significance by unpaired *t*-test for mean scores at different days after immunization. **c** Area under the curve (*AUC*) was calculated between days 0 and 33 post-immunization (*p.i.*) (D0–33), and differences between groups were analyzed using the Mann–Whitney test; standard error of the means are shown; n = 16 mice/group. **d** Disease incidence at day 20 p.i. was analyzed by Fisher’s test. The bar graphs for representation of the disease incidence contingency table show the numbers of symptomatic and asymptomatic mice at day 20 p.i. for each group. *p < 0.05, **p < 0.01, ***p < 0.001

We also tested different post-MOG inoculation approaches, once the peripheral immune response had been triggered. Administration of DMSCs once the mice had reached EAE scores higher than 2.5 failed to yield any beneficial effects (data not shown). However, continuous administration of DMSCs to MOG_35–55_-primed mice with lower scores showed that DMSCs could also limit the disease progression when the immune response had already been triggered. For the experiment depicted in Fig. 9, the mice received the first DMSC dosage at day 6 p.i. or at day 10 p.i. (with score values higher than 1), and four additional doses were administered every 3–4 days. For as long as DMSC continued to be dispensed, the clinical signs were significantly milder for both treated groups as compared to the EAE control group (Fig. 9a and b). In addition, the AUC showed lower values for the DMSC-treated animals (Fig. 9c). Moreover, as for the preventive treatment, disease incidence at day 20 decreased by DMSCs, even if treatment had begun when the disease symptoms were already developed (Fig. 9d).

## Discussion

The results of this study, showing that EAE is significantly ameliorated by human decidua-derived MSCs (Figs. 1, 8 and 9), suggest that these cells could be seen as a promising cell therapy for MS. We describe here that in vivo treatment of EAE mice with DMSCs inhibits T-cell proliferation driven by antigen presentation and downregulates IL-17 production (Fig. 3). The role of IL-17 in the severity of EAE has been extensively demonstrated [3, 4]. It is likely that the decrease in IL-17 secretion after DMSC treatment is related to impaired differentiation of CD4^+^ cells into the Th17 phenotype, since expression of the master regulator for Th17 development, RORγT, is downregulated in DMSC-treated animals. This view is reinforced by data from cocultures of T cells with DMSCs, showing diminished percentages of IL-17^+^ and RORγT^+^ cells after TCR stimulation (Fig. 5). In spite of the fact that Th17 and Foxp3^+^ Treg cells are CD4^+^ subsets mutually exclusive [68], we did not find differences between treated and untreated EAE mice in the levels of Foxp3 mRNA in CD4^+^ cells nor in the percentages of spleen CD4^+^Foxp3^+^ cells (Figure S1 in Additional file 1). However, we cannot rule out that other regulatory cells, as myeloid-derived suppressor cells, could be involved in the lack of proliferation of spleen cells from DMSC-treated EAE mice (Fig. 3).

On the other hand, DMSCs were able to inhibit Th17 establishment even in the Th17-skewing culture conditions generated by IL-6 and TGFβ. Suppression of Th17 cells during EAE by BM-MSCs has also been observed [14, 24, 32], and a recent report has shown that placenta-derived adherent cells led to diminished numbers of IL-17-producing cells in spinal cord infiltrates [41]. Our data from experiments with DMSC-conditioned medium suggested that the inhibiting activity of the Th17 differentiation process is mediated by one or more soluble factors produced by DMSCs (Fig. 6). In contrast, a cellular cross-talk requirement has been reported for the attenuation of IL-17 expression on T cells by BM-derived MSCs [69]. Such differences in the mechanisms involved in Th17 control might be linked to the MSC source. Most probably this discrepancy could be related to the different maturation state of T cells. Ghannam et al. [69] analyzed the effect of MSCs on fully polarized Th17 cells, whereas we studied the behavior of T cells during the Th phenotype polarization. Indeed, Luz-Crowford et al. [70] found that BM-MSCs displayed different mechanisms, based on soluble factors or on direct cell interaction for regulation of IL-17 production during Th17 development or in fully polarized Th17 cells, respectively.

Concomitant with the reduced levels of IL-17, splenocytes from DMSC-treated animals produced higher levels of IL-4 and IL-10 than did EAE control mice (Fig. 3). Such cytokines have a critical role in tolerance induction, resistance to, and recovery from, EAE [8, 9, 71, 72]. Judging by the results of the analysis of the master transcription factors and the cytokine profiles in isolated CD4^+^ spleen cells, DMSCs act directly on this cell type, resulting in a deviation of the Th phenotype in favor of the Th2 versus Th17 subset. However, as IL-4 and IL-10 are not solely produced by CD4^+^ cells, DMSCs could also induce other spleen cell types to produce them. In fact, DMSC-induced increase in IL-10 levels was more noticeable in total spleen cell cultures than in isolated CD4^+^ cells, which might suggest that, besides CD4^+^ cells, other cell populations could also be induced by DMSCs to deliver this anti-inflammatory cytokine. Similarly, other studies in different disease animal models have reported IL-4 or IL-10 increases by MSCs from different sources [14, 24, 37, 41, 73, 74].

In addition to the Th2 phenotype deviation in peripheral immune organs during DMSC treatment of EAE, we found lower numbers of infiltrating IL17^+^ cells in the spinal cord of treated animals (Fig. 7). We cannot currently discern whether this difference in cellular infiltration is due to reduced migration to the CNS or to a direct effect of DMSCs on the target organ. Regardless of the cause for the decreased IL17^+^ cell infiltration in the CNS, it could affect the recruitment of CD11b^+^Ly6G^+^ neutrophils, as indeed was found by the cell composition analysis of CNS infiltrates (Fig. 7). A main role for IL-17 is to cooperate in the chemoattraction of polymorphonuclear leukocytes, mainly neutrophils, to inflammatory sites through induction of CXCL-8, CXCL1 and CXCL2 [75–77], which is critical for the disruption of the blood–brain barrier [60]. Alternatively, the diminished number of neutrophils in inflammatory infiltrates could be due to a direct effect of DMSCs unrelated to IL-17 levels. In this regard, some immunomodulatory properties of MSCs have been ascribed to secretion of TSG-6 [78, 79], a molecule recently involved in inhibition of neutrophil migration by interaction with CXCL-8 [80]. We also detected a reduction in CD11b^+^Ly6C^+^ cell infiltration in the CNS of DMSC-treated animals. CD11b^+^Ly6C^+^ cell subtype, which identifies inflammatory monocytes [58], is another typical component of CNS infiltrates in EAE [64, 66]. This myeloid subset constitutes the precursor of macrophages and dendritic cells able to produce high levels of tissue damage mediators, such as TNFα and IL-1 [58]. These also act as antigen-presenting cells, reactivating T cells to contribute to the inflammatory cascade in the CNS. A reduction in the number of neutrophils and mature macrophages has been described in MSC therapy for other disease models, such as traumatic brain injury [81], ischemia [82], acute kidney injury [83], and allergic inflammation induced by *Aspergillus* [84]. However, to our knowledge, there are no previous reports on MSC treatment of EAE showing diminished presence of neutrophils or monocytes in inflammatory infiltrates. No effect of DMSCs was found in the number of spleen neutrophils or monocytes (data not shown), suggesting that a migration deficiency might underlie the diminished presence of these cell types in the CNS. Any or all of the immunomodulatory effects triggered by the DMSC treatment described here could contribute to a less pro-inflammatory environment in the target organ, which might be involved in the restraining of tissue damage observed in DMSC-treated mice (Fig. 2).

DMSCs offer several advantages over MSCs from other tissues to be used as a therapy, such as easy isolation of cells without any invasive procedures. DMSCs are of maternal origin but express factors involved in pluripotency and organogenesis though not embryonic markers (SSEA-1, -3, -4 and TRA-1-81) [42] expressed by other placental-derived MSCs [46]. These features allow high plasticity and differentiation capacity into derivatives of all germ layers, with reduced ethical problems with respect to embryonic stem cells. In addition, DMSCs also display high genomic stability after proliferation in culture, and low and limited telomerase activity [42, 43, 45]. DMSCs, like BM-derived MSCs, do not express the major histocompatibility complex class II nor the T-cell costimulatory molecules, conferring them an intrinsically hypo-immunogenic and immunomodulating stem cell character [41, 42, 45]. These properties would allow DMSCs to be tolerated, potentially effective and clinically useful in allogeneic receptors. Supporting this discernment, MSCs derived from healthy, full-term human placentas have been administered to relapsing-remitting and secondary progressive MS patients via intravenous infusion. The results showed that placental MSC injections in these patients were safe and well tolerated [23]. In addition, DMSCs show higher proliferation rates and differentiation capacity than do BM-derived MSCs [47, 48]. Furthermore, DMSCs can be easily cryopreserved over the long term without losing their original phenotype, exponential growth, and differentiation characteristics. Indeed, all the effects of DMSCs on EAE described above were observed with thawed cryopreserved cells. Thus, based on the high proliferation rate of placental-derived MSCs, a single donor could be used for multiple patients after cell storage. Finally, these maternal-derived mesenchymal stromal cells could be also an autologous source for cell therapy on several diseases developed by the mother [42]. All these advantages make DMSCs a potentially safe and useful product for future use in humans.

Our results show effective prevention of the disease through short-term DMSC treatment, albeit of limited durability. Interestingly, periodical administration of the cells results in mild clinical course of the disease, even if animals receive the first treatment when they are already presenting moderate symptoms (Figs. 8 and 9). Along with preliminary studies with autologous BM-derived MSC transplantation in MS patients yielding promising results, the clinical use of alternative cell sources, such as placenta-derived DMSCs, warrants further investigation to address whether the long-term safety and potential clinical efficacy of using this new cell therapy could actually provide improvements over other sources of MSCs. Since MS is a chronic disease, several authors have suggested that, to ensure a sustained therapeutic benefit, clinical application of MSCs might require repeated administration of cells instead of just one dosage [18, 29]. MSCs are thought to function through a ‘hit-and-run’ mechanism, whereby they release an array of cytokines and trophic factors without any significant engraftment. Under conditions of chronic autoimmunity and CNS injury, multiple doses of MSCs are likely to be necessary for the sustained production of immunomodulatory and trophic factors in order to exceed a therapeutic threshold [29]. This scenario is in line with our results in EAE mice, which strongly support a cell therapy strategy based on repeated administration of DMSCs, by adapting the dose schedule to the clinical level, which might substantially modulate the long-term progression of MS, the main treatment goal in this progressive and disabling demyelinating disease.

## Conclusions

This study reveals immunomodulatory effects of DMSCs able to modulate EAE. The beneficial properties of such treatment on the disease signs, and on the frequency in infiltration foci in the CNS, correlate with impairment of the Th17 phenotype in favor of the promotion of IL-4 and IL-10 production at the early immune response. Therefore, human decidua seems to be a valuable source of MSCs, with therapeutic potential for MS and for other immunological diseases in which IL-17 has a pathogenic role.

## Additional files

Additional file 1: Figure S1. Foxp3 expression by T cells from untreated or DMSC-treated EAE mice (n = 9/group). Spleen cells from EAE animals were obtained at day 10 p.i. Total RNA samples were obtained from purified CD4^+^ cells and used to quantify Foxp3 mRNA by RT-qPCR (A). Total spleen populations from individual mice were analyzed by cytometry analysis of surface and intracellular staining with anti-CD4-APC and anti-Foxp3-PE, respectively (B). Percentages of CD4^+^Foxp3^+^ cells are shown by representative flow dot plots and by the average of the values obtained for each individual mouse from each group. Standard error of the means are shown. (PDF 150 kb) Additional file 2: Figure S2. In vitro treatment of CD4^+^ T cells with DMSCs interferes with Th17 phenotype definition. CD4^+^ cells purified from C57BL/6 mice spleens were stimulated in vitro by anti-CD3/anti-CD28 antibodies under nonpolarizing condition (A) or under pro-Th17 pressure in the presence of IL-6 and TGFβ (B). Three-day cultures were analyzed for IL-17 expression. Soluble IL-17 measurements were quantified by ELISA. Each sample was assayed in quintuplicate and significance was analyzed by *t*-test; standard error of the means are shown. Results are shown from one representative out of three independent experiments. (PDF 17 kb)

## Abbreviations

AUC: Area under the curve
BM: Bone marrow
CNS: Central nervous system
D: Day
DMEM: Dulbecco’s modified Eagle medium
DMSC: Decidua-derived mesenchymal stem cell
EAE: Experimental autoimmune encephalomyelitis
EGF-1: Epidermal growth factor 1
ELISA: Enzyme-linked immunosorbent assay
IFN: Interferon
IL: Interleukin
LFB: Luxol fast blue
MOG: Myelin oligodendrocyte glycoprotein
MSC: Mesenchymal stem cell
MS: Multiple sclerosis
PAS: Periodic acid-Schiff
PBS: Phosphate-buffered saline
p.i.: Post-immunization
RT-qPCR: Retrotranscription and quantitative real-time polymerase chain reaction
TGF: Transforming growth factor
Th: T helper
TNFα: Tumor necrosis factor-alpha
Treg: T regulatory
