# Supplementary figures and images for: Restrained Th17 response and myeloid cell infiltration into the central nervous system by human decidua-derived mesenchymal stem cells during experimental autoimmune encephalomyelitis
Source: Stem Cell Res Ther. 2016 Mar 17;7:43. doi: 10.1186/s13287-016-0304-5 (PMC4797118; doi:10.1186/s13287-016-0304-5)

A

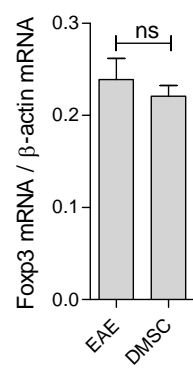

B

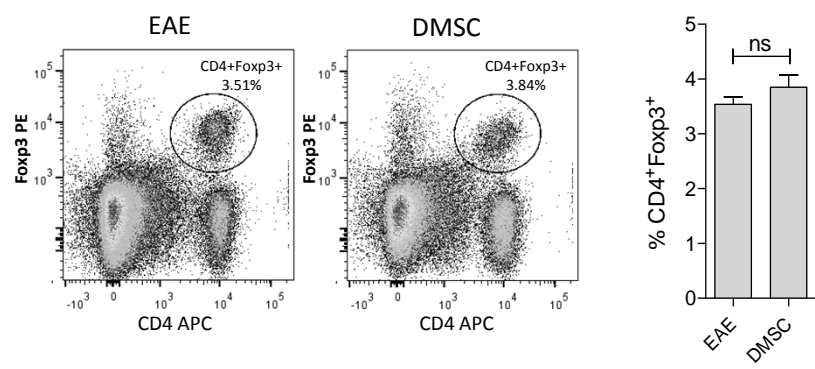

Supplement: Additional file 1: Figure S1. — Foxp3 expression by T cells from untreated or DMSC-treated EAE mice (n = 9/group). Spleen cells from EAE animals were obtained at day 10 p.i. Total RNA samples were obtained from purified CD4+ cells and used to quantify Foxp3 mRNA by RT-qPCR (A). Total spleen populations from individual mice were analyzed by cytometry analysis of surface and intracellular staining with anti-CD4-APC and anti-Foxp3-PE, respectively (B). Percentages of CD4+Foxp3+ cells are shown by representative flow dot plots and by the average of the values obtained for each individual mouse from each group. Standard error of the means are shown. (PDF 150 kb) [file 13287_2016_304_MOESM1_ESM.pdf]

**A**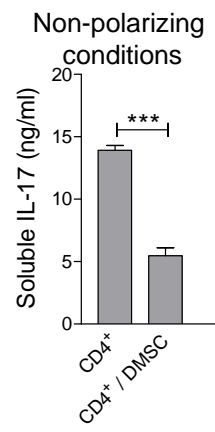

First  $\alpha$ CD3/ $\alpha$ CD28 round

**B**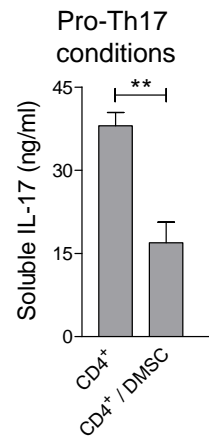

Supplement: Additional file 2: Figure S2. — In vitro treatment of CD4+ T cells with DMSCs interferes with Th17 phenotype definition. CD4+ cells purified from C57BL/6 mice spleens were stimulated in vitro by anti-CD3/anti-CD28 antibodies under nonpolarizing condition (A) or under pro-Th17 pressure in the presence of IL-6 and TGFβ (B). Three-day cultures were analyzed for IL-17 expression. Soluble IL-17 measurements were quantified by ELISA. Each sample was assayed in quintuplicate and significance was analyzed by t-test; standard error of the means are shown. Results are shown from one representative out of three independent experiments. (PDF 17 kb) [file 13287_2016_304_MOESM2_ESM.pdf]
